# The Medical Association of Georgia

**Published:** 1884-05

**Authors:** 


### Society Proceedings.

---

### THE MEDICAL ASSOCIATION OF GEORGIA.

The thirty-fifth annual session of the Medical Association of Georgia, convened in Macon on April 16th, and continued in session three days.

### FIRST DAY.

The Association was called to order by the retiring President, Dr. K. P. Moore of Macon. After prayer by Rev. A. J. Battle, D. D, Dr. Wm. F. Holt of Macon, in behalf of the Committee of Arrangements, delivered the following address of welcome :

*Mr. President and Gentlemen of the Medical Association :* The city of Macon but welcomes her own once more when she extends to the Medical Association of Georgia a sincere and cordial greeting. Thirty-five years have passed, years fraught with the vicissitudes of sorrow, suffering and renewed prosperity to many of our Southern homes, since in this city and in a hall across yonder street the Medical Association of Georgia was called into existence. In looking over this assembly, I note only a few of those who were present at that meeting and assisted in its organization. They will give you whispers from the store houses of their memories; will tell you with what serious doubts and misgivings, with what anxious solicitude they watched its growth and progress during the early years of its existence, how at times its early demise was seriously threatened, and confidently predicted, what incessant care and earnest attention were necessary to nourish the constantly flickering and oft-times nearly extinguished flame of the little nursling's life. But to-day, thanks to the unremitting exertion of the profession, we behold it full grown, strong and vigorous, an institution worthy of our veneration snd support.

It is then, gentlemen, with peculiar pride and pleasure that upon this the thirty-fifth anniversary of its existence, and in this city of its birth, that in behalf of the Committee of Arrangements and the medical profession of Macon, I extend to you a cordial greeting and a hearty welcome to our city and to our homes. Seven years have elapsed since we had the pleasure of greeting you in our midst. We are glad to see you and expect much pleasure from your visit, and sincerely wish each and all of you to feel at home—at liberty to go when and where your inclination prompts you.

While we cannot show you a harbor filled with vessels, floating the representation of sails of almost every nation on the globe, nor to a canal bearing on its turbid waters the products of the country, nor to a State house (that some of us think ought to be here) with its massive blocks of granite and marble. yet we do claim to possess the educational city of Georgia—a city of literary culture, of colleges and schools.

We will point you to Wesleyan—the oldest female college in the world—with its magnificent building and commanding location—a building, handsome not only in its exterior, but perfect in its internal arrangements and especially adapted to the health and comfort of the pupils; Mount de Sales, Appleton Home, Mercer University, with its distinguished Alumni, Georgia Academy for the Blind, Pio Nono College, Orphan Home, numerous private schools, and then to our public schools, located in every section of the city, where an education can be obtained alike by the rich and the poor.

Each and all we would cordially invite you to visit, feeling assured that you will find much to interest and amuse you.

With the hope that the present meeting will be a pleasant and harmonious one—one that will redound to the honor and interest of the entire profession—and that you will carry with you pleasing recollections of your sojourn with us, I bid you heartily welcome.

Dr. L. B. Alexander, of Forsyth, on behalf of the Association, responded as follows:

*Mr. President*: I arise, sir, in behalf of the Medical Association of the State of Georgia to respond to the address of welcome and generous tender of the hospitalities of the city of Macon so handsomely offered by Dr. Holt.

In performing this pleasant duty I am pleased to say that we accept the offer with a hearty good will.

Some of us who have labored long and earnestly in our noble calling have known Macon long and well. We knew her before these stately walls of brick and mortar were erected. We knew her before she numbered half her present inhabitants. But, sir, we never knew her when she was not full of generosity, full of hospitality, full of energy and full of enterprise. Macon has always done her duty, and done it well and wisely.

Within a very recent period of time she has increased her borders, has pulled down her old structures and erected larger and more handsome ones in their places. Her numbers have doubled, and her territory of trade has reached out and out until she has become a commercial centre of strong proportions. Her magnitude of solid wealth has outstripped her most sanguine expectations.

While we would say this much and more of your proud city, we do not forget to say that the brotherhood of our noble profession here have kept pace with her progress and prosperity.

Not many years since you had in your midst a Harrison, a Greene, a Boone, a Pye, a Burgess, and a Nottingham. These were a band of noble brethren, but they have long since gone to their reward, while their bodies sleep quietly and peacefully beside the banks of the ever-flowing Ocmulgee. But you still have prominent with you a Hammond, a Fitzgerald, a Holt, a Hall, a Stephens—and recently Moore has come amongst you, and a score of others of younger years who are able and willing to illustrate our noble calling.

Ours, sir, is a noble calling. It is second to none save the ministry. We feel that it was begun by Christ himself. Did he not by the aid of some anæsthetic agent cause a deep sleep to come upon Adam while he performed the first surgical operation? Did he not minister to the sick? Did he not restore the palsied limb? Cleanse the leper? Cause the lame to walk? The dumb to speak and the deaf to hear? Yea, verily, and from him it has been handed down from generation to generation all along through the ages until to-day, now in the nineteenth century, we find ourselves following along in his footsteps, riding up and down through the earth. going from house to house, closing up the wounds, ministering to the afflicted, and I may add, binding up the broken-hearted.

We, sir, have gathered here from all parts of the State. We have come from the mountains, where the pure crystal fountains flow to make glad the weary, worn traveler in search of a better and a purer atmosphere—we have gathered from the beautiful Savannahs, where the magnolia and the bays load the air with their delightful fragrance. We have come from our Southland, where the plains stretch out in every direction. We have come from amongst the "Old Red Hills," where peace and contentment reign supremely. Yes, sir, we have come from all parts of our noble old State and have gathered ourselves together as with one mind and heart to receive knowledge and impart instruction. We have come for the advancement of science and art. We have come not only to discuss questions of material interest to our brotherhood, but we have come to see you, to shake hands with you and sit down in pleasant places to reason together as brethren. Yes, sir, we have come to do all these and more; we have come to eat your bread, and to drink your wine, and to look upon the faces of your beautiful women, who, like so many precious jewels, glitter all over the bosom of your beautiful Hill City. They are God's last, best and noblest gift to man, and the poet has fittingly said that:

> They were formed to bless the life of man
> And share his care,
> To soothe his breast when keen distress
> Hath lodged a poisoned arrow there.

In conclusion now, sir, I will say that for this three days' session we are *yours* truly.

Dr. K. P. Moore, of Macon, the retiring President, introduced the President-elect, Dr. A. W. Calhoun, of Atlanta, in the following address:

"Sixteen years ago, when I left the fostering care of my alma mater, and cast my frail barque upon the turbulent stream of a professional life, I felt like one upon a broad and expanseless sea without chart or compass. In the chilly darkness of that stormy period I naturally looked out for some friendly lighthouse along the shore. Across the breaking waves came the gleaming light of the Medical Association of Georgia; and to her inviting haven I bent my oars. Under her watch care I was kindly received, and for fifteen years I have annually looked forward to her coming sessions with pleasurable anticipations of no ordinary character. During those years the meeting with familiar faces, and the re-unions which I have enjoyed, have been to me genuine annual love feasts. During those years, ties of friendship have been formed so strong and lasting, that nothing will ever break, save the last great wave which shall lash each one of us in our turn upon the endless shores of eternity. During those years the association has several times seen fit to place me in positions of honor and trust, and now, in retiring from the highest gift of honor within the gift of her membership, it is no meaningless expression of formality, when I return to you my most grateful acknowledgements for many kindnesses and courtesies of which I have been the honored recipient. To say that these many expressions of confidence have awakened within my bosom emotions of deepest and most profound gratitude is but a faint expression of my real feelings.

When the history of our country shall have been fully written, each and every trade and profession will point with pride to some illustrious names which shall adorn the pages of that history. It gives me peculiar pleasure to introduce to you to day. as my successor in office, one in whom the medical profession of Georgia feels a just and special pride—one, the mention of whose name brings emotions of gratitude to many a heart throughout this broad sunny land of ours; one through whose skillful touches the dark shades of a long, dreary night, have been driven away, and through the visual windows the beautiful scenes of a new-born world have been let in upon many a benighted soul – a ripe scholar, a cultured gentleman and a public benefactor, in honoring whom the Medical Association of Georgia has honored itself—Dr. A. W. Calhoun, of Atlanta, Ga."

Dr. Calhoun arose and addressed the Association as follows:

*Gentlemen of the Medical Association of Georgia—Ladies and Gentlemen:* It will perhaps surprise some of you that I have departed from the usual order of addresses before this Association, and to some degree have been actuated by rather a selfish motive in the selection of a subject, for I have fallen upon one not only in accord with my own tastes, but one which I feel assured will impress each individual of this intelligent audience with its appropriateness, with its practical facts, and with its notes of timely warning.

My subject—School Hygiene in Relation to its Influence upon the Vision of Children, or School Sanitation—does not express the full scope of this discourse, but is a faint outline of what I shall have to say to you in the time allotted to me.

Education is the preparation for the work of life, not a thing that is good in itself. If it has helped life to be healthy, happy, successful and long, then it has been good; if in any degree it has caused disease, unhappiness, non-success, then it has been bad. The medical aspects of life are deservedly attracting more and more the attention of doctors in every land where education and civilization have made much progress. The researches of one investigator developed the fact that out of more than 2,300 infants examined by him, only 122 possessed abnormal peculiarities of any kind, and from this fact drew the conclusion that children, as a rule, are physically sound when they start to school. That deformities of one organ or another, simple or serious, do exist amongst school children admits of no argument, and many of these may be reasonably attributed to the influences surrounding a child during its life in the school room. The various parts of the organism of youth are easily disarranged, and if the cause operates continuously the disarrangement is liable to become permanent.

Before the child ever sees the inside of a school room he learns many useful things—often more than he does in the next succeeding five years—and yet this learning does not injure his eyes. It is a fact that children at the time of entering school are free from that common affection, near-sightedness, but almost immediately thereafter the disease begins to show itself. This is conclusive evidence that the fault to a great degree, at least, lies in the school.

Before his school days begin, and while the child is learning, and that too very rapidly, all the knowledge that he gains is real knowledge of concrete things, gained through the use of all his senses and all his activities by being brought into contact with the things about which he learns. He is free to sit still or to move; to fix his attention upon a thing as long as it interests him, and then to leave it for something else. But at school nearly everything is unnatural. Very often the child is seated on a hard bench, so high that his feet can not touch the floor, with the back, if it have any, so straight that a comfortable position is impossible, and the desk, if there be any so far in front of him that he can not use it without leaning forward much further than is good for either his back or his eyes; and there with foul air to breathe, with windows improperly shaded, or not at all, with light coming from the front as often as otherwise, and with little or nothing to make the place look cheerful or homelike, he is confined for six hours a day, five days in the week, from twenty-eight to forty-five weeks in the year, and for as many years as his constitution can stand such abuse, or his parents afford to send him. In this room he seldom comes in contact with natural things, or even with representations of them, excepting his teacher and fellow students, and they, too, are made as unnatural as possible. Seated on his high, hard bench, prohibited from looking to the right or left, book in hand, he is committing to memory the words of the author, and this they call getting an education. "What's in a name."

It has been truthfully said that sight is the noblest avenue of the mind, and its impairment or loss is a greater evil than would be that of any other bodily sense. For many years deterioration of the eye among pupils in public and private schools has been a subject of complaint and investigation both in this country and the older portions of the world This impairment of sight, this deterioration of the eye, is principally the immediate result of Myopia, or nearsightedness which, in its turn, is caused by defective modes of education and their hurtful surroundings.

Myopia is a disease. A near-sighted eye is not a normal eye. Children born in the normal state do not have near-sighted eyes. But what is myopia? Anatomically the difference between a normal eye and one that is myopic, or near-sighted, is practically a difference in the length of the eye-ball The slightest fractional increase in the length of the ball beyond its normal length is an increase of myopia. As the eye becomes elongated the retina, which receives impressions, and upon which images are formed, is thrown beyond the focus of the rays of light coming from a distance.

Very rarely before the fifth or sixth year of life does myopia make its appearance, about which time children usually begin their attendance upon school but from this time on, under certain unfavorable circumstances, the eye gradually elongates, reaching and remaining, perhaps, at a certain point of elongation, a slight or high degree, or constantly increasing through all the years of school life, even to the twenty-fifth year, and indeed in some instances, continuing slowly to lengthen through almost the whole of life.

Myopia is essentially a disease of childhood, beginning from the sixth to the fifteenth year, just at a time when the body as a whole is developing most rapidly. Rarely does it originate after the twentieth year. When once in existence in a child, it is usually progressive, and therein lies the danger; and this fact, coupled with the fact that it is an incurable disease, makes it an important subject in connection with education, for the causes that produce the disease at this period of life also operate to increase it. The eye of a child is a plastic organ, easily changed in its shape, and its tissues are in a condition to be readily modified by the use which is made of the organ. The child goes on to the eighth or tenth year, perhaps a little longer, when it is observed that it has to hold whatever it is looking at a little nearer to the eye than previously, and then, upon examination, the fact is revealed that the eye is myopic or near-sighted. If you follow such a child up to the age of twenty-five or thirty years, it will be found that the myopia has doubled, and perhaps quadrupled.

I cannot here refrain from quoting a well known author, who thus writes:

'Predisposition to myopia is almost always inherited by at least some of the children where one parent is myopic. In these children myopia may often be detected, if sought for, at a very early age, and is generally evident at from eight to twelve years of age. Once present, it tends to increase, and should be watched with care. If not existing at least in some degree before sixteen years of age it is never developed, even by excessive use of the eyes.

"During the period of youth, which is usually also the time of closest application to study, there is a disposition to gradual development of the inherited myopic tendency; but this may be kept in abeyance if the eyes are used principally for large objects; and if, during this period, the myopia does not become very considerable, it may remain stationary during the rest of life. Temporary increase of myopia may take place during these years of growth and of study from too close application; but, provided its degree is still moderate, its further progress may be arrested at or after maturity if the individual grows more prudent. But, and this constitutes the gravest feature of the disease, if the myopia has, during this period, already reached a high degree, the tendency to continued progress frequently cannot be arrested, notwithstanding the exercise, too late, of the greatest care; and degenerative changes go on in the tissues and media of the eye, with the sad prospect of partial or even total blindness at or before middle age.

"Since it has been shown that it is especially by continued tension of the muscle of accommodation in study that myopia with its attendant dangers is generated and increased, and as it is well known to be only preventable and not curable, it follows that a change in our methods of education is an absolute necessity, or else this which might be termed self-imposed disease, will impose a more and more grievous burden on the community.

"A child having an hereditary leaning towards myopia is expected to give a large portion of time every day to study of oftentimes badly printed books, perhaps in a dim light, and sometimes with the requirement from his teacher that he shall not take his eyes from his lesson. Myopia is thus begun. As this augments, the child who does not see things about him clearly, has less pleasure in the usual sports of his age, and finds more enjoyment in books. His close application to reading, writing, drawing. etc., keeps up convergence of the eyes and pressure upon them of the recti muscles, which tends little by little to increase the ellipsoid change of form and elongate the antero-posterior axis. These alterations go on during the period of growth and of most continuous study, because at this time the tissues of the globe are softer and more extensible than after maturity. If on reaching this latter term the structural changes are still only moderate in degree, the myopia may continue stationary during life. But if at this time great deviations from the normal condition have already been produced, the affected parts are less capable of resisting further yielding, and progressive myopia is thenceforward an ever-present source of danger.

"Prevention is the sole resource at our command; restoration is impossible. And in order that preventive measures may be seasonably adopted, it is first necessary that the profession and the public should become alive to the fact that in a large number of cases myopia is one of the gravest affections of the eye, capable of limitation by constant care during childhood and youth; but, if not thus limited likely to be a source of future disability and misery, and to be handed down as an onerous inheritance to children. At present the warnings inspired by frequent sad experience in the practice of every skilled observer are almost unheeded, and it is but too common to see the chances of retaining even moderately useful vision in future years recklessly sacrificed to a vain ambition for acquiring mere book knowledge, which, when gained, is often valueless to its possessor, or if otherwise it could be usefully applied, cannot be made serviceable because of the imperfection of sight which has been created in obtaining it.

"Very high degrees of myopia should also be recognized as an infirmity deserving careful consideration before assuming the obligations of marriage; for those in moderate circumstances may well hesitate to choose partners who, though highly cultivated, may probably at middle life become unable to provide for their households or their children."

That the eye grows weaker as the term of study grows longer, is clearly proven by actual investigation. Careful tests have been made of the eyes of many thousand school children in America, Germany, Austria, Russia, Switzerland and other countries, and invariably with similar results, viz: that the proportion of normal-sighted children generally lessens as the age of the subjects advance, and as they reach the higher grades of study. The statistics thus gathered show that while in children at common village schools there is but one-fourth of one per cent. of myopia, it rises to 21 per cent. in city schools of high grade, and in some high schools and universities it has reached 60 to 70 per cent. The examination of six hundred students of theology at the University of Tuabingen found 79 per cent. suffering from myopia. Other statistics have shown that in those who studied two hours out of school, the proportion of myopic students was 17 per cent.; in those studying six hours 40 per cent. Germany furnishes more myopic, or near-sighted subjects than any other country in the world, her schools showing at least 62 per cent., while in America, so far as examinations extend, the rate is about 27 per cent. So it would seem that even in our own land the school-room is a factor most directly influential in the gradual and increasing development of a race of spectacle using people. The ratio has been found to be smaller in America than in Europe, because, probably, of the greater activity and variety of life and the less degree, as yet, of hereditary tendency. A curious fact made known by these investigations is, that colored school children suffer so slightly from myopia as to be practically free from it.

These facts deserve serious attention, especially in connection with our undoubted power of modifying or arresting the progress of myopia by proper management and by self-denial during the years of growth and of education.

Myopia, says one, is especially prevalent among the so-called cultivated classes, and the more time people spend in intellectual pursuits the more myopia do we find. Yet students do not use their eyes for more hours a day, or on finer objects than jewelers, engravers, draftsman, seamstresses, type-setters, and many others who engage in long continued work on small objects. These occupations do not show any tendency to myopia, while the professional and literary callings do. The particular reason why members of mechanical arts show less myopia than those of studious and literary occupations, is not because they use their eyes less, but that the application of their eyes occurs at a different time of life and under entirely different conditions. Germany is confessed one of the most studious nations in the world, and she certainly is the most near-sighted. Many of her school houses are very old structures, originally built for convents, and poorly lighted; moreover, the German text itself is obscure compared with the clear Roman letters.

In writing and study, it is easier to sit bending over an ordinarily located desk, in the stooping position, than in an erect posture. This position prevents by compression the free return of blood from the head. The posture of the head favors its detention in the eyes, while the working of the brain itself demands more blood, and hence we easily have a congestion of the eye-ball, and especially of those parts that are most active, viz: the retina and optic nerve. With this congestion there occurs softening of the sclerotic or outer coat and increase of the fluid contents of the eye-ball, increasing the pressure from within. At the same time, the muscles on the sides of the ball, produce pressure without in their effort at converging for real vssion. This condition of things causes a bulging of the posterior wall of the eye, and in this way myopia, or near-sightedness begins and increases, for the same causes continue to act with greater force as the trouble progresses. In children the tissues of the eye-ball are much softer than in the adult, and this is one of the principal reasons why these causes are more active in producing the disease between the ages of six and twenty. Undoubtedly the disease is often hereditary, but the predisposition to it may be largely counteracted by proper care. The absence of myopia among savages is attributable to absence of hereditary tendency together with absence of undue tension of the eyes for near objects. British surgeons tell us out of many thousands of the natives of British India examined by them, not a single near-sighted one was found. Who of you can call to mind one near-sighted negro who lived in *ante-bellum* days? Education has put its mark upon them now, however, and wonderful to relate, they are proud of their infirmity.

The examination of several hundred negro school children in New York found only 2½ per cent. mvopic In many negro schools in the South, examinations discover less than 1 per cent. Absence of hereditary predisposition largely explains this difference, but a few generations hence, with increased educational facilities, and with the hereditary tendency steadily increasing, near-sightedness will become not an uncommon disease among negro students.

Bad air and defective light play an important role in the production of myopia. Says a distinguished author: "Air and light are the first and last and best messages of life—the first breath and the last breath – the first glance and the last glance; how wonderful!"

Bad air alone, in a school room, acting as a primal cause, may set in train a series of morbid processes, which may, and often do, affect not only the working capacity and integrity of the organ of vision, but which may lead even to its total destruction. One of the professors in the law school at Cambridge, in commenting upon the frequency of eye diseases in that institution, says, it must not be supposed that the young men injure their eyes by excessive application. "Bad ventilation and the gas-heated air of the lecture room cause the trouble."

But light, plenty of good light, is one of the chief needs of the scholar. Too much light can never be thrown into the school-room, especially when we have at our command the means of regulating the excess of glare. A room is not sufficiently lighted when a child cannot easily read fine print, on a moderately clear day, at a distance of twelve to fifteen inches. The less the light, the nearer an object must be brought to the eye, and the greater the strain in the act of vision; for reduction in illumination is, as a rule, precisely equivalent to a reduction in the size of the object. A model school room would be one in which there was not only an abundance of good light and of good quality, but in which the eyes both of pupils and teachers were alike shaded from the painful glare. In all cases the light should come from the left and at a distance of four to five feet from the floor. Next to this a rear light is permissible, but light from the right should, if possible, never be used. Windows should never be placed in front of the pupil. Light from such a direction is positively injurious. They should always be placed on the left side; the next best place is the rear and after this the right side, which position should only be occupied when no other can be obtained. But the true light should come from over the left shoulder. It does not harm the eye, does not cast a shadow on the pupil's work and is not reflected directly into the eye. A German writer thus sums up the results of his investigations: "The narrower the street in which the school house was built, the higher the opposite buildings, and the lower the story occupied by the class, the greater the number of near-sighted scholars."

A recent investigator writes: "Among the causes of visual weakness among American youths may be named a stooping posture, which cramps the chest and brings the eye too near the book or paper; reading at twilight and late at night, and studying by lamp light in the early morning, reading in the cars, using kerosene lamps without shade, reading while facing a window or any light natural or artificial, and still more while facing the bright sunshine, reading dime novels or other books printed in too fine type, reading while lying in bed, wearing a veil; and neglecting to cultivate far-sightedness by carefully examining distant objects. Hence myopia is more common in cities than in the country, more among those working on near and minute objects than those laboring in the fields with a wider range of vision and more objects to invite habits of observation."

Tension of the accommodation, that is, long continued use of the eye upon objects brought close to it, is considered by all authorities one of the most (if not the most) fertile causes of progressive near-sightedness. The act of reading involves very considerable physical labor. It is said a book of 500 pages, 40 lines to the page and 50 letters to the line, contains 1,000 000 letters, all of which the eye has to take in, identify, and combine each with its neighbor. Yet many readers will go through such a book in a day. The task is one he would shrink from if he should stop to measure it beforehand.

It is well known to every investigator that imperfect type is influential in the production of eye diseases. "Bright white paper, particularly if its surface is glazed, is dazzling or irritating. It is on account of the quality, rather than the size of English print, that it is usually so much pleasanter to read than American. 'Some cheap publications manage to combine all of the defects referred to, in such a degree that a more paternal government than ours might well suppress them as enemies of society. Fortunately, such publications do not contain intellectual treasures that it need tempt one to risk his eyes to reach." While too fine print is regarded as a factor in bringing about eye disease, it must not be forgotten that to ocoarse print is wearisome to the eye, for it requires more exertion of the muscles govering the movements of the ball. Especially is this the case if the breadth of the page is too great. It is for this reason that the narrow form of the English blank verse is so little fatigueing to the eye. A double-column page which is well printed and properly divided, is certainly preferable to the same amount of matter extending in a single line across the entire page.

An enthusiastic and ingenious writer asserts that "nature and science declare" that the color of the paper of all books should be green. "Green grass covers the ground and green leaves are our canopy, and no other color is so grateful to the eye. Let our books be printed on green paper, and let our printers use red, yellow or white ink for the noxious black." When this has been done, he says, "everybody will rejoice except the spectacle makers. The eyes of the scholar and of the student will no longer be wearied with the myopian contrast of black and white, but strengthened and refreshed by congenial colors; and to pour over the pages of a book would be no more fatigueing to the eyes than gazing on a verdant prairie decorated with variously tinted flowers" We must agree with him in this, that the reform would be revolutionary, and that the interest of the trade would be hostile to the change.

The best safeguards against harm are afforded by the best positions and best light, clear type, plain inks, with the best paper cf yellowish tints and abundant space between the lines.

" The increased demand that the exigencies or the fashion of the times make upon the eyes as well as upon the brains of the children, and the increased numbers that are yearly brought within the influence of school life by the compulsory laws of governments or of public opinion, should be accompanied by a corresponding increase in the use of all the alleviations and precautions that science and humanity can suggest. School training is necessarily an artificial process, and unless it is conducted under rational and favorable conditions, universal education can never be an unmixed universal blessing." Much of the injury to body and mind as well as sight is traceable to causes which goad the children on to tasks that the brightest and strongest of them are scarcely equal to, and the "higher education" that is now so earnestly demanded for the gentler sex, is too often dearly bought at the expense of shattered constitutions and ustrung nerves. But if these things must be, in the name of humanity and justice, let them be surrounded by all the checks that can lessen their power for evil.

After a diligent study upon the question of construction of school houses and the construction and arrangement of desks and seats, a distinguished surgeon has stated, that 90 per cent. of curvature and other diseases of the spine are developed during and largely attributable to school life. It should not be forgotten that "there is an architecture for schools as well as an architecture for palaces. One is not less worthy of study than the other, and we are at fault in taste as well as in hygiene if we forget that here real beauty consists above all things, in the perfect adaptation of a building to its uses." An abundance of properly regulated light, good ventilation and plenty of room, are the essentials of a perfect school house.

A certain school committee after thorough investigation, reports that it is "of the opinion that the practice of requiring pupils to commit their lessons from books, is not only the cause of much of this near-sightedness, but that it is a most pernicious practice from every point of view, and more especially so when much of the so-called studying must be done at home evenings. Children under fourteen or fifteen years of age, should never be required to get regular lessons out of school hours. This is not to say they must spend those hours out of school in idleness or play."

Fewer hours of study and more rational methods of teaching, less cramming of mere memory and more healthy development of the intellect, will make brighter, healthier and more intelligent students, who will develop into stronger, hardier, more energetic men and women. For such as these the practical affairs of life offer many and varied fields of usefulness, while for the sallow-faced, narrow-chested, weak-eyed, book worm, there is no room in a busy world.

After the President's adddress, the Secretary read letters and telegrams from various absent members expressing regrets at not being present.

When the reports of Special Committees were called for, the Secretary read a letter from Dr. Jos. P. Logan, of Atlanta, Chairman of the Committee on Inebriate Asylums, stating that every effort had been made to have such an institution established, but had failed, and that he was satisfied further effort was unnecessary, and asked that the committee be discharged This request was granted.

Dr. H. V. M. Miller, of Atlanta, made a verbal report as Chairman of the Committee on expert testimony. He said that he was satisfied that the views of the Association in regard this subject could not be secured by legislation. He stated that the members of the Legislature regarded any action in that direction as special legislation, and would not, therefore, lend their support. He, at the same time. submitted a verbal report as Chairman of the Committee on Anatomical Material.

He stated that a bill had been prepared and presented to the Legislature providing for Anatomical material. and that the bill passed both branches of the Legislature and failed to become a law, the Governor vetoing it after the Legislature adjourned. He stated that the question stood in the main as it did before the introduction of the bill. He recommended that the committee be continued and instructed to persistently urge the next Legislature to pass some law providing anatomical material for dissecting purposes. The committee was continued.

The Secretary submitted the report of the Committee of Publication, which was referred to the Auditing Committee, consisting of Drs. Eugene Foster, R J. Nunn and W. B. Wells.

Dr. Eugene Foster of the Committee on Necrology, presented touching and beautiful memorial tributes on the lives and characters of Drs. E. W. H, Hunter, of Louisville; Sterling Eve, of Augusta; J. M. Carlton, of Athens, and L. D. Ford, of Augusta. The last was specially feeling and earnest and was received with utmost attention and reverence by the Association. Report adopted.

Committee on Prize Essays, Dr. Foster, Chairman, said that no prizes had been awarded, inasmuch as the committee did not deem any of the essays received of sufficient merit.

When the reports of committees from the various Congressional Districts were called, Dr. R. J. Nunn announced report on Gynecology, from the First District. The Doctor gave a synopsis of the report and exhibited a number of new instruments and appliances devised by himself

Dr. N. P. Jelks submitted a report on Practice of Medicine from the Third District.

---

### SECOND DAY.

The morning session was devoted chiefly to the reading of reports of Sections from the various districts.

At 12 o'clock the President, Dr. A. W. Calhoun, introduced the orator of the day, Dr. Mark H. O'Daniel, of Milledgeville, who addressed the Association in a neat and well-timed address. In concluding Dr. O'Daniel said:

" And of all this, gentlemen, what is the object and what is the end? None other than the discovery of truth and the application of this truth to suffering humanity. Such are the aims of him who enters into the right spirit upon the study of our science, and engages in the practice of our art. Can there be a nobler combination than that practice opens to our view—the intellect keenly laboring for the benefit of our fellow-men, and the affections deeply sympathizing in the results of the labor?

" May not a class of men who are devoted to the task of warding off that grim monster, death, of shortening the career of diseases, of assuaging physical pain and of smoothing the passage to the grave, I say, may not this class of men lay claim to an elevated position in the social scale? Ought it not be a high privilege to belong to a profession of which this is the exalted mission?

"It is not vividly transpiring. Ought it not in itself to suffice to cheer us on amid toil, amid neglect, amid ingratitude, amid worldly struggles, and last, but not least, amid poor remuneration, to remember that by taking a position in its ranks, we have acquired the power to think, to feel, to act for the accomplishment of things so great, that we have insured for ourselves the enjoyment of pleasures so pure. But if our profession confers such privileges and supplies such foundation for nobler orders of happiness, a return is looked for on part of us who bear the name. We should be worthy and high-minded members, should maintain its dignity, elevate its position as far as our individual character, conduct and acquirements can conduce to that end. Our moral feeling and character should be of such high order as to hold us beyond the reach of malignity. This is expected of medical men, and it is fitting and just it should be.

"A punctilious attention to the principles of nicest order should uniformly guide us in intercourse with one another. We should all be true, love one another, the profession with which we are honored and advocate, work together in the future as in the past with untiring efforts for its future development and advancement, until the diseases, the cure of which now puzzles the brightest minds of our profession, shall take wings and fly from appliances and discoveries made at our hands.

"And now, gentlemen, allow me to close this address with words of well-wishing to you all, and with the hope, that we may all meet again at our next annual meeting, and cause it to be more interesting and profitable than ever before.

"Pursue the sacred counsels of your soul, which urge you on to virtue; let not danger nor the encumbering world, make faint your purpose. Assisting angels shall conduct your steps, bring you to bliss and crown your end with peace."

At the conclusion of the orator's address, the Association adjourned to 3 o'clock.

AFTERNOON SESSION.

Dr. V. H. Taliaferro, of Atlanta, submitted a report on Gynecology from the Fifth District. The Doctor exhibited a number of plates and drawings illustrating his report.

Dr. W. B. Wells, of Red Clay, submitted a report on Surgery for the Seventh District, which he asked be referred to the Committee on Publication, without reading. This request was granted, and the report will go to the Committee on Publication.

Dr. T. M. Holmes, of Rome, submitted a report on Practice for the Seventh District. Referred to the Committee on Publication.

Dr. John Gerdine, of Athens, submitted a report on Practice of Medicine, which was referred to the Committee on Publication.

When the call for voluntarily papers was made, the following were read by title to be called up and read at the pleasure of the Association:

By Dr. A. G. Hobbs, of Atlanta, "Jequirity—its use in the treatment of granular lids."

By Dr. H. J. Williams, Macon, " A case of empyæmia successfully treated by free incisions, constant drainage and antiseptic injections—remarks.

By Dr. J. M. Hull, Augusta, " Extreme age, no contra indication for cataract extractions with cases."

By Dr Eugene Foster, Augusta, "Syphilis as a socio-logical problem —the opinions and statements of Mr. Herbert Spencer thereon reviewed."

By Dr. J. P. Stevens, Macon, " Animal Fermentation "

By Dr. W. B. Parks, Atlanta, " A new mammary Bandage."

By Dr. J. W. Flanders, Wrightsville, " Successful Removal of Uterine Tumor per Vergenum."

By Dr. J. W. Flanders, Wrightsville, " A Case of Supposed Superfœtation."

By Dr. J. W Flanders, Wrightsville, "A Case of Immediate Resection of the Humerus with Union and Subsequent Legthening of the Bone."

By Dr. H. McHatton, Macon, "Propagation of Leprosy."

By Dr. H. F. Scott, Atlanta, "Tobacco Amblyopia. with cases."

By Dr. N. G. Gewinner, Macon, " Treatment of the Placenta after Abortion "

By Dr. F. Eklund, of Stockholm, presented through Dr. H. F. Campbell, of Augusta, "Observations on the Essential Nature of Diabetes Mellitus Vulgaris "

By Dr. E. G. Ferguson, Macon, "Nature's Laws."

By Dr. C. W. Hickman, Augusta, " Mitral Insufficiency."

By Dr. J. McF. Gaston, Atlanta, " Explanation of the Pathology and Therapeutics of the Nerve Centres—Especially Epilepsy."

By Dr. T. M. McIntosh, Thomasville, "Cases in Surgery."

By Dr. H. V. M. Miller, Atlanta, "The Effects of Altitude in the Treatment of Consumption."

By Dr. W. O'Daniel, Bullards, "Plaster-Paris Apparatus in the Treatment of Fractures.

By Dr. W. F. Westmoreland, Atlanta, "Should we use Plaster of Paris Splint in fractures accompanied with contusions and lacerations immediately after Injury."

By Dr. K. P. Moore, Macon, Ga., "A Plea for a more general use of Anæsthesia in Natural Labor, with some Physiological Suggestions upon the rationale of its action as a reason therefor."

By Dr. W. C. Gibson, Macon, Ga., "Two cases of Cilia Blepharits, associated with Simple Hypermetropia and cured by correcting the Hypermetropia with Proper Glasses."

By Dr. Robert Battey, Rome, Ga., "Antiseptics in Ovariotomy and Battey's Operation. Thirty cases without a death."

The nominating committee reported for President, Dr. Eugene Foster, of Augusta; for first Vice-President, Dr. J. B. Roberts, of Sandersville; second Vice-President, Dr. W. D. Bizzell, of Atlanta.

Adopted.

There was no election for Secretary and Treasurer, their term of office not expiring until 1887.

---

### THIRD DAY.

The Association was called to order promptly at 10 o'clock A. M., by the President.

Dr. Wm. A. Love, of Atlanta, a member of the Committee on Necrology, submitted memorial tributes of Dr. Alexander Means, of Oxford; Dr. Thomas Raines, of Atlanta, and Dr. McMillian, of Columbus.

Dr. John Thad. Johnson, of Atlanta, reported a very interesting case of Hydrophobia, which he saw some month's previous. Dr. Hull, of Augusta, reported several cases that had come under his observation.

On motion, the Association requested Dr. Miller to read the paper reported by title the day previous.

The paper was read and commanded marked attention. We refrain from giving a synopsis of this valuable paper for the reason that it will soon appear in THE JOURNAL.

Through its chairman, Dr. J. T. Johnson, the Committee on Nominations reported the following committees for the various Congressional districts:

First District—Section on practice, W. Duncan, chairman. Section on surgery, W. H. Elliott, chairman. Section on gynecology, R. J. Nunn, chairman.

Second District—Section on practice, T. S. Dekel, chairman. Section on surgery, A. P. Taylor, chairman. Section on gynecology, T. S. Hopkins, chairman.

Third District—Section on practice, S. B. Hawkins, chairman. Section on surgery, R. H. Pate, chairman. Section on gynecology, T. F. Walker, chairman.

Fourth District—Section on practice, C. D. Smith, chairman, Section on surgery, W. A. Pool, chairman. Section on gynecology, W. W. Fitts, chairman.

Fifth District—Section on practice, W. D. Bizzell, chairman. Section on surgery, W. P. Nicolson, chairman. Section on gynecology, J. G. Earnest, chairman.

Sixth District—Section on practice, L. B. Alexander, chairman. Section on surgery, T. H. Kenan, chairman. Section on gynecology, T. O. Powell, chairman.

Seventh District—Section on practice, W. S. Kendrick, chairman. Section on surgery, W. B. Wells, chairman. Section on gynecology, Robt. Battey, chairman.

Eighth District—Section on practice, J. Gerdine, chairman. Section on surgery, G. W. Mulligan, chairman. Section on gynecology, J. E. Pope, chairman.

Ninth District—Section on practice, J. W. Bailey, chairman. Section on surgery, W. J. Rusk, chairman. Section on gynecology, L G. Hardman, chairman.

Tenth District—Section on practice, S. D. Brantley, chairman. Section on surgery, A. G. Whitehead, chairman. Section on gynecology, H. F. Campbell, chairman.

The report was received and adopted.

The President then announced as the next annual orator, Dr. J. B. Baird, of Atlanta.

Dr. Eugene Foster submitted his report, as chairman of the auditing committee. Among other things they reported that:

"We have examined the books and statements of the Treasurer and of the Secretary and find them to correspond with the vouchers accompanying them.

"We desire to draw attention to the energy displayed by the Secretary in procuring a number of advertisements, without which, owing to the delinquency of members of the Association, they could not have published their proceedings.

"We also wish to express our satisfaction at the neatness and accuracy with which the accounts of the Treasurer and Secretary have been kept."

Adopted.

Interesting papers were read by Dr. H. J. Williams, of Macon, and Dr. J. McF. Gaston, of Atlanta The paper of Dr. Gaston was especially interesting, and we regret we have not space to give a more extended notice of it now.

The President announced the following delegates to the American Medical Association :

Drs. Eugene Foster, H F. Campbell, Jos. P. Logan, A. G. Whitehead, G. W. Mulligan, H McHatton, B. R. Dostor, De S. Ford, W. H. Doughty, Sr., W. F. Westmoreland, V. H. Taliaferro, W. O'Daniel, K. P. Moore, A. J. Logan, C. D. Smith, T. O. Powell, R. J. Nunn, T. J. Charlton, Robt. Battey, W. B Wells, Jno. Gerdine, E. Fitzgerald, M. G. Hatch, W. P. Nicolson, P. L. Hilsman, J. C. Soloman, James A. Gray, S. B. Hawkins, G. G. Crawford, T. M. McIntosh, E. C. Goodrich, T. R. Wright, C. H. Hall, A. W. Calhoun, G. W. Holmes, E. L. Connally, J. S. Todd.

On motion, the Presidents name was added to the list.

The President then appointed the following Committee on Arrangements for the next meeting: Dr. J. G. Thomas, chairman ; Dr. Dr. J. P. Read, Dr. T. J. Charlton, Dr. J. C. LeHardy, Dr. W. Duncan. All of the gentlemen appointed were from Savannah.

The following resolution offered by Dr. T. F. Walker, was unanimously adopted :

"*Resolved*, That in consideration of the elaborate report of the entire proceedings of the Association having from day to day been published in the *Macon Telegraph* and *Messenger*, making a perfect record for all the members to read at their leisure, that the thanks of this Association be extended to said newspaper and to the faithful reporter, Mr. Jule Rodgers, who has been persistent in his attendance and efforts to obtain a correct and satisfactory report."

Adopted.

On motion the President, Dr. A. W. Calhoun, in a few pointed words, adjourned the Association to meet in Savannah on the third Wednesday in April, 1885.

We regret exceedingly that want of space forbids our mentioning more of the excellent papers that were read and discussed.

NOTES.—The Association is placed under lasting obligations to the physicians of Macon, for the royal hospitality bestowed upon it during its stay in their beautiful city.

The grand banquet given on Thursday night was unsurpassed by anything of the kind ever tendered the Association.

The writer of this is placed under special obligations to Drs. Wm. F. Holt, K. P. Moore and H. J. Williams, of Macon, for favors shown.

